# Artificial Intelligence in Dental Education: A Scoping Review of Applications, Challenges, and Gaps

**DOI:** 10.3390/dj13090384

**Published:** 2025-08-25

**Authors:** Mohammed El-Hakim, Robert Anthonappa, Amr Fawzy

**Affiliations:** University of Western Australia, Dental School, Perth, WA 6009, Australia; robert.anthonappa@uwa.edu.au (R.A.); amr.fawzy@uwa.edu.au (A.F.)

**Keywords:** artificial intelligence, dental education, machine learning, simulation, assessment, AI-assisted learning

## Abstract

**Background/Objectives:** This scoping review aims to map existing AI applications in dental education, in student learning, assessment, and diagnostic training, identifying key limitations and challenges. **Methods:** Following the Arksey and O’Malley framework and PRISMA-ScR guidelines, six databases were searched in March 2025 using combinations of the following search words: “dental education,” “artificial intelligence,” “machine learning,” and “student assessment”. Inclusion was limited to English-language empirical studies focused on dental student education. Of 547 identified studies, 17 met the inclusion criteria. They were categorized into four domains: (1) Preclinical Training, (2) AI in Clinical, Diagnostic Training, and Radiographic Interpretation, (3) AI as an Assessment Tool and Feedback System, and (4) AI in Content Generation for Dental Education. **Results:** AI has positively influenced various domains, enhancing procedural accuracy, diagnostic confidence, assessment efficiency, and content delivery. However, it struggles to assess nuanced competencies like dexterity and clinical judgment. The challenges faced include disparate definitions of AI, ethical and privacy concerns, model variability, and a deficiency of dental leadership in AI development. At present, most tools are engineered by computer scientists and may not align effectively with the priorities of dental education. **Conclusions:** AI holds significant potential to enhance dental education outcomes. However, to guarantee its relevance and reliability, it requires standard frameworks, ethical oversight, and clinician-led development. Future research should concentrate on implementing real-time AI-driven feedback systems during preclinical training and advocate for more precise definitions to support consistent AI application and evaluation in dental education.

## 1. Introduction

Dental education is rapidly evolving to include new technologies that prepare students for modern clinical practice. Artificial intelligence (AI) has emerged as a transformative tool in health professions education, offering new approaches to teaching and decision-making. AI is broadly defined as the simulation of human intelligence by systems capable of perception, reasoning, learning, planning, and prediction [1,2]. In medical and dental education, AI applications such as radiographic analysis, diagnostic simulations, and automated assessments have demonstrated early promise. However, the systematic integration of AI into dental curricula remains underdeveloped [3].

The adoption of AI in dental education can enhance learning through personalized feedback and objective assessments. AI-powered systems may increase procedural accuracy, strengthen radiographic interpretation, and streamline grading processes. These innovations align with the shift toward competency-based, student-centred education [3,4,5].

Despite its potential, AI implementation in dental education faces challenges, including concerns about reliability, faculty readiness, and a lack of standardized frameworks. [6,7] Furthermore, ethical concerns around data privacy, bias, and responsible AI use require attention within the educational context [8,9,10]. Many faculty members report limited AI knowledge and insufficient training, despite expressing positive attitudes toward AI integration [7,11].

To ensure consistency and clarity, this review adopts a standardized definition of AI as the simulation of human intelligence by machines capable of perception, reasoning, learning, planning, and prediction [2]. This definition aligns with established technological frameworks and distinguishes AI from other educational tools. AI research revived in 2006 with Hinton’s deep learning model, enabling machines to learn autonomously [2]. This definition guided the review’s evaluation of AI in dental education.

Virtual reality (VR) is an example of a misclassification of AI in dental education research due to the lack of a standardized definition. While VR supports simulation, it lacks adaptive learning and autonomous decision-making, key features of true AI [12]. Clearer definitions are needed to avoid such inaccuracies and ensure consistency in AI-focused studies.

This scoping review aims to identify, summarize, and categorize existing applications of AI in dental education, with a focus on student learning, assessment, and diagnostic training. Additionally, it seeks to explore current limitations and challenges, providing insights to guide future research and support the responsible integration of AI into dental curricula.

## 2. Materials and Methods

This review followed the five-stage scoping framework proposed by Arksey and O’Malley [13], further refined by Peters et al. [14]. This scoping review was conducted and reported in accordance with the PRISMA-ScR (Preferred Reporting Items for Systematic Reviews and Meta-Analyses extension for Scoping Reviews) 2020 guidelines [15]. The review protocol was not registered. In line with standard scoping review methodology, no formal critical appraisal of included studies was performed, as the aim was to map the breadth of available literature rather than assess study quality.

However, two reviewers independently assessed each study for potential bias based on study design, sample size, outcome measures, and presence of control groups. Bias was categorized as low, moderate, or high to aid interpretation. Discrepancies were resolved by consensus, and ratings are presented in Table 1.

### 2.1. Research Question

Using the Population, Concept, and Context (PCC) framework [16], the guiding scoping question was: What are the applications, limitations, and challenges of artificial intelligence in dental education?

### 2.2. Search Strategy

PubMed, Web of Science, Cochrane Library, Google Scholar, Dentistry & Oral Sciences Source, and Embase were searched in March 2025 using combinations of the following search words: “dental education”, “artificial intelligence”, “machine learning”, and “student assessment”. Included studies applied AI empirically in preclinical, clinical, or assessment-based dental education.

### 2.3. Eligibility Criteria

Inclusion:

- English-language empirical studies
- Direct evaluation of AI in teaching, feedback, or dental student assessments

Exclusion:

- Opinion pieces or perception-based surveys
- Studies unrelated to AI or dental education
- Reports on AI solving exams or general curriculum reform
- Studies not directly related to the education of dental students or their teaching

**Figure 1:**
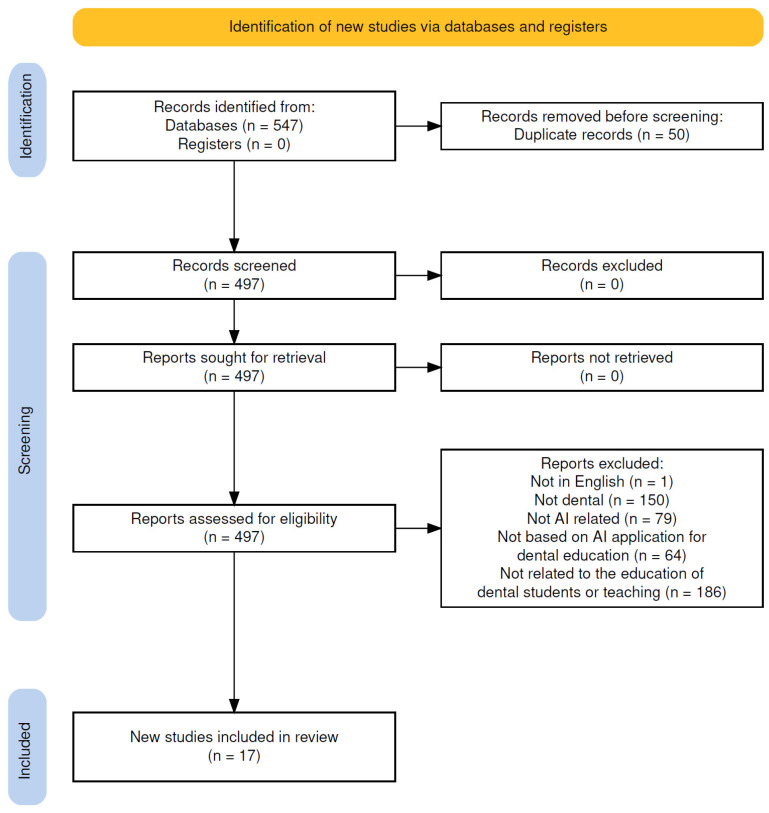
PRISMA flow diagram [17] for the scoping review process performed.

A total of 547 records were identified through database searches. After removing 50 duplicates, 497 records were screened. Following the application of inclusion and exclusion criteria, 17 empirical studies were selected for inclusion in the review. Two reviewers assessed the full texts of all 497 articles. They reached an agreement on excluding 480 studies due to reasons such as lack of empirical data, absence of focus on dental students, or classification as commentary or editorial pieces. The complete study selection process is illustrated in the PRISMA flow diagram (Figure 1). All studies that met the eligibility criteria during full-text assessment were included in the final synthesis.

### 2.4. Data Charting and Synthesis

Data were extracted from the included studies using a structured Excel-based (Microsoft 365) data charting form developed by the review team. The author extracted that data and presented it to the review team with key information from each study, including author, year, country, aim, design, sample size, AI domain classification, key findings, limitations, and bias risk. Discrepancies between reviewers were resolved through discussion and consensus. All necessary information was available in the published reports. No data transformations or conversions were required. All data were extracted as reported in the original studies and summarized descriptively. Missing numerical details were not imputed, and all synthesis was based on the available published information. Results of individual studies were tabulated in a structured summary table (Table 1), organized by domain, with key information including study aim, design, strengths, limitations, findings, conclusions, and bias risk rating. This table was developed to support thematic synthesis and comparison across domains. A descriptive, narrative synthesis was used to summarize and categorize findings across the included studies. This approach was chosen due to the heterogeneity of study designs, AI applications, and outcome measures, which precluded quantitative synthesis. Thematic grouping by domain allowed for structured comparison and identification of trends, challenges, and research gaps. No statistical meta-analysis was performed. No statistical methods were used to explore heterogeneity among study results, such as subgroup analysis or meta-regression, as this scoping review aimed to map the scope of evidence rather than quantify effects across comparable studies. No sensitivity analyses were conducted, as the aim of this scoping review was to map the breadth of existing literature rather than evaluate the robustness of effect estimates through quantitative synthesis.

Risk of bias was assessed qualitatively by both reviewers during full-text screening and data extraction. We considered methodological transparency, clarity of research aims, sample size, and alignment between findings and conclusions. Due to the heterogeneity of study designs, no formal risk of bias tool was used. Additionally, risk of bias due to missing results or selective reporting was not formally assessed, as this scoping review did not involve outcome-level synthesis or effect estimation. Certainty in the body of evidence was also not assessed, given the review’s aim to map the scope and characteristics of existing literature rather than to evaluate the strength of evidence for specific outcomes.

Extracted data were categorized into four domains based on thematic analysis of the included studies. As no standardized classification framework existed for AI in dental education, we developed our own domain structure. Two reviewers independently reviewed each study’s objectives, methods, and outcomes, and allocated them to the most relevant domain through consensus. Where studies overlapped multiple domains, classification was based on the dominant theme. This ensured consistency in synthesis and interpretation across domains.

1. Preclinical training [18,19]
2. AI in Clinical, Diagnostic Training, and Radiographic Interpretation. [20,21,22,23,24,25,26,27,28,29]
3. AI as an Assessment Tool and Feedback System [30,31,32]
4. AI in Content Generation for Dental Education [33,34]

## 3. Results

Seventeen peer-reviewed studies met the inclusion criteria. They were published between 2022 and 2025. Findings are summarized by domain:

### 3.1. AI in Preclinical Training

AI simulation platforms improved procedural accuracy, student confidence, and feedback quality. However, no study included real-time feedback on operative procedures such as cavity preparation, highlighting a current research gap. The two included studies in this domain used quasi-experimental and observational designs with moderate risk of bias. Both relied on self-reported student perceptions and had relatively small sample sizes. Despite positive findings, limited generalizability and lack of objective outcome measures reduce certainty.

### 3.2. AI in Clinical, Diagnostic Training, and Radiographic Interpretation

AI has shown strong potential in dental education by improving diagnostic accuracy and supporting clinical decision-making. Tools like chatbots and image analysis systems enhance pattern recognition and often outperform student assessments. However, limitations in context, ethics, and the need for human oversight mean AI should support, not replace, clinical judgment. This domain included the largest group of studies, with varied designs including randomized trials and comparative analyses. Risk of bias ranged from moderate to low. Studies with expert benchmarks and objective diagnostic outcomes showed stronger methodology, while those using perception-only data or small samples had higher bias risk.

### 3.3. AI as an Assessment Tool and Feedback System

AI is used in dental education for automated grading and real-time feedback, enhancing efficiency and reducing bias. It supports personalized learning by identifying student weaknesses and offering adaptive guidance. However, AI struggles with evaluating complex, subjective responses and lacks the depth needed for holistic clinical judgment. Ethical concerns, lack of standardization, and student over-reliance highlight the need for faculty oversight and careful integration. Studies in this domain explored AI-generated feedback and grading but primarily relied on small samples and student self-reporting. They were judged to have a moderate risk of bias due to limited outcome validation, short study duration, and potential novelty bias.

### 3.4. AI in Content Generation

AI is being used to generate educational content in dental education, including case studies, quizzes, and interactive modules, enhancing efficiency and accessibility. Studies show AI tools can support self-directed learning and improve information retrieval compared to general models. However, faculty oversight is needed to validate accuracy and ensure content meets accreditation standards. Key challenges include potential bias, limited nuance in complex cases, and ensuring student engagement. Both studies in this domain were early-stage or pilot evaluations of AI-generated educational content. Risk of bias was rated as moderate due to limited external validation, absence of student outcome assessment, and potential inconsistencies in AI-generated material.

### 3.5. Key Findings

AI plays a significant role in enhancing various aspects of dental education. In preclinical training, AI has been shown to improve learning outcomes by supporting decision-making and boosting student confidence. It also enhances diagnostic training, particularly in radiographic interpretation, by supporting more accurate and consistent image analysis. Automated grading to improve efficiency, though it often lacks human-like feedback for deeper learning.

### 3.6. Identified Gaps

Several key gaps remain in the integration of AI within dental education. There is a lack of research on AI’s role in real-time procedural assessment, such as evaluating restorative cavity preparation or restoration accuracy, leaving its potential in preclinical feedback, interactive clinical training, and preclinical student assessments underexplored. Additionally, AI models designed for interactive, case-based diagnostic reasoning are still underdeveloped. Current AI grading systems also fall short in providing contextual, human-like feedback, which is essential for supporting meaningful learning and reflective practice.

### 3.7. Challenges

Across the reviewed studies, several key challenges to integrating AI into dental education were identified. A major concern is the inconsistent application of AI definitions, highlighting the need for clearer terminology and guidelines. Over-reliance on AI poses risks, as it cannot replace the critical thinking and clinical judgment fundamental to dentistry. Ethical and privacy issues arise from patient data use, demanding strong governance frameworks. Successful integration further depends on faculty training and institutional support. Progress is additionally limited by the lack of dental leadership in AI development, as most models are created by computer scientists, reducing alignment with dental education priorities such as preclinical skills training.

**Table 1:** Summary of Included Studies Categorized by AI Domain in Dental Education.

Category of AI Domain Classified by This Scoping Review	Author, Year, Location	Aim	Design	Strengths	Limitations	Findings	Scoping Review Conclusions
AI in Preclinical Training	Choi et al. (2023), Australia [18]	To develop and evaluate an interactive AI system for assessing student performance in endodontic access cavity preparation and provide immediate feedback.	Observational study; 79 fourth-year dental students participated, but only 44 completed the post-intervention evaluation survey.	Development and implementation of a novel AI assessment system Real-time, individualized feedback for skill refinement Use of a structured Likert-scale survey to gather student feedback	Only 44 out of 79 participants completed the post-survey (55.7%) Subjective self-reported feedback, no objective skill performance measure Single-center study limits generalizability	Students found the AI feedback system helpful for identifying and correcting errors High satisfaction was reported for system usability and self-directed learning support Most students preferred combining AI feedback with instructor feedback	The AI-based feedback system was positively received and promoted student engagement in access cavity preparation. However, further validation is required using objective outcome measures and control groups. Bias Risk: Moderate due to reliance on self-reported outcomes, and incomplete survey responses.
AI in Preclinical Training	Mahrous et al., 2023 USA [19]	To compare student performance in removable partial denture (RPD) design using traditional methods versus the AiDental AI and game-based learning software, and to assess student perceptions of the AI tool.	Quasi-experimental study; two-group comparison (AI-game group vs. control group); *n* = 56 (28 students per group).	Blinded assessment of practical exam Integration of AI and gamified learning Direct evaluation of student perception	Sample size: 73 students (*n* = 36 intervention, *n* = 37 control) Short intervention period (2 weeks before testing) Survey-based perception data without long-term follow-up	The AiDental group outperformed the control group in RPD design accuracy and completeness (statistically significant improvement). Survey results indicated positive student perception, with high ratings for engagement and usefulness.	The integration of AI and game-based learning improved short-term performance in RPD design and was well-received by students. However, the short duration and reliance on a single institution limit generalizability. Bias Risk: Moderate due to the short intervention period, absence of long-term outcomes, and reliance on self-reported perceptions.
AI in Clinical, Diagnostic Training, and Radiographic Interpretation	Or et al., 2024, Australia [20]	To evaluate the feasibility and effectiveness of an AI-powered chatbot for improving patient history-taking skills among dental students.	Pilot observational study using a single cohort of third-year dental students interacting with an AI chatbot for simulated history-taking.	High engagement: 100% student participation with the chatbot compared to 2/13 in traditional tutorials Realistic simulation using GPT-3.5-based patient responses Scalability and accessibility for repetitive practice	Small sample size (*n* = 13 students) No control group or pre-post performance measures Subjective outcome assessment via perceived usefulness	Students reported increased engagement and perceived improvement in competence after using the chatbot Staff supported the tool’s educational value and future use potential Highlighted potential for expanding to early-year students and other case types	The chatbot was positively received and showed promise as a supplementary tool for dental education. However, due to the small sample size and lack of control group, findings must be interpreted cautiously. Bias Risk: High, due to subjective measures, small sample, and lack of comparative analysis.
AI in Clinical, Diagnostic Training, and Radiographic Interpretation	Aminoshariae et al., 2024, USA [21]	To explore how artificial intelligence (AI) can be integrated into endodontic education and identify both its potential benefits and limitations.	Scoping review of 35 relevant studies, conducted through electronic and grey literature search across databases including MEDLINE, Web of Science, and Cochrane Library up to December 2023.	Comprehensive literature search strategy including grey literature and ongoing trials. Categorized AI applications into 10 key educational areas, providing structured thematic synthesis. Inclusion of multi-institutional and international author expertise.	No quality appraisal of included studies, limiting the ability to weigh strength of evidence. Possible selection bias due to broad inclusion criteria and lack of systematic review methodology.	Identified 10 domains where AI may enhance endodontic education: radiographic interpretation, diagnosis, treatment planning, case difficulty assessment, preclinical training, advanced simulation, real-time guidance, autonomous robotics, progress tracking, and educational standardization. AI supports individualized feedback, structured simulation, and automated decision support.Emphasizes that integration of AI will shift the traditional pedagogy of Endodontics.	AI holds promise to revolutionize endodontic education through personalized learning, diagnostic assistance, and simulation-based training. However, educators must acknowledge its current limitations and ensure its responsible implementation. Bias risk: Moderate due to lack of critical appraisal of included studies and variability in evidence quality across reviewed articles.
AI in Clinical, Diagnostic Training, and Radiographic Interpretation	Ayan et al., 2024, Turkey [22]	To assess the diagnostic performance of dental students in identifying proximal caries lesions before and after training with an AI-based deep learning application.	Randomized experimental study involving pre- and post-testing of two student groups, one receiving AI-based training.	Use of a validated deep learning algorithm (YOLO—You Only Look Once) tailored for caries detection. Expert-labeled dataset (1200 radiographs) ensures strong ground truth reliability. Comparative pre- and post-intervention design increases internal validity.	Small participant sample size (*n* = 40 dental students). Only one institution involved, limiting generalizability. Increased post-test labeling time in AI-trained group may indicate increased complexity or cognitive load.	AI training led to statistically significant improvement in accuracy, sensitivity, specificity, and F1 scores (*p* < 0.05). No significant difference in precision score. Labeling time increased in the AI-trained group.	Training with AI significantly enhanced students’ ability to detect enamel and dentin caries on radiographs. Although longer labeling time was observed post-training, the educational benefit suggests that AI can serve as a valuable tool for radiographic interpretation training. Bias Risk: Moderate due to small sample size, single-site recruitment, and lack of long-term follow-up.
AI in Clinical, Diagnostic Training, and Radiographic Interpretation	Chang et al., 2024, USA [23]	To evaluate (1) the efficiency and accuracy of dental students performing full-mouth radiograph mounting with and without AI assistance, and (2) student perceptions of AI’s usefulness in dental education.	Randomized controlled experimental study with two student groups: manual vs. AI-assisted radiograph mounting. Pre- and post-study surveys were also administered.	Real-world clinical simulation with third-year students. Inclusion of both objective (time and accuracy) and subjective (student perceptions) outcome measures. Random allocation of 40 participants enhances internal validity.	Small sample size (*n* = 40 dental students). Single institution study. AI assistance led to reduced accuracy, suggesting issues with over-reliance on automation. No long-term assessment of retention or skill transfer.	AI-assisted group completed radiograph mounting significantly faster (*p* < 0.05). However, the AI-assisted group demonstrated significantly lower accuracy than the manual group (*p* < 0.01). Student confidence and perceptions of AI did not differ significantly between groups, before or after the intervention.	While AI assistance improved efficiency, it negatively impacted accuracy, indicating that premature automation may hinder skill development in novice learners. Students maintained neutral perceptions toward AI, highlighting the need for careful integration of AI tools in early dental education. Bias Risk: Moderate due to small sample size, lack of blinding, and single-site scope.
AI in Clinical, Diagnostic Training, and Radiographic Interpretation	Prakash et al., 2024, India [24]	To develop and evaluate *DentQA*, a GPT3.5-based dental semantic search engine aimed at improving information retrieval for dental students, while addressing issues like hallucination, bias, and misinformation.	Tool development and validation study using both non-human (BLEU score) and human performance metrics, including accuracy, hallucination rate, and user satisfaction.	Combines objective (BLEU score) and subjective (human evaluation) performance assessments. Tailored specifically to dental education content. Compared directly to GPT3.5 baseline for benchmarking. Evaluated on 200 questions across multiple categories.	Total of 4 human evaluators only and 200 questions evaluated. Lack of real-world classroom or clinical implementation. Evaluations limited to document-based Q&A rather than broader clinical decision-making tasks.	*DentQA* outperformed GPT3.5 in accuracy (*p* = 0.00004) and significantly reduced hallucinations (*p* = 0.026). Demonstrated consistent performance across question types (*X^2^* = 13.0378, *p* = 0.012). BLEU Unigram score of 0.85 confirmed linguistic reliability. High user satisfaction with an average response time of 3.5 s	*DentQA* provides a promising AI-based solution for reliable and efficient information retrieval in dental education. Its reduced hallucination rate and consistent performance across question types support its potential as a domain-specific educational tool. Further testing in real academic settings is recommended. Bias Risk: Moderate due to a limited number of evaluators and absence of practical deployment data.
AI in Clinical, Diagnostic Training, and Radiographic Interpretation	Qutieshat et al., 2024, Oman [25]	To compare the diagnostic accuracy of dental students (junior and senior cohorts) with that of a modified ChatGPT-4 model in endodontic assessments related to pulpal and apical conditions, and to explore the potential role of AI in supporting dental education.	Comparative observational study using seven standardised clinical scenarios. Diagnostic accuracy was measured for junior and senior dental students versus ChatGPT-4, with expert-derived gold standards as reference.	Included a large student sample (*n* = 109). Included junior (*n* = 54) and senior (*n* = 55) groups for subgroup analysis. Used gold-standard expert assessments for accuracy comparisons. Applied robust statistical analysis (Kruskal-Wallis and Dwass-Steel-Critchlow-Fligner tests). Clearly delineated performance metrics for AI vs. students.	Scenarios limited to seven predefined cases may not generalize across broader clinical complexity. No evaluation of long-term retention or educational impact post-AI exposure. Does not assess student reasoning or process—only final answer accuracy.	ChatGPT-4 achieved 99.0% accuracy, outperforming senior students (79.7%) and juniors (77.0%). Median diagnostic accuracy: ChatGPT = 100%, Seniors = 85.7%, Juniors = 82.1%. Statistically significant difference between ChatGPT and both student groups (*p* < 0.001). No statistically significant difference between senior and junior groups.	AI, specifically ChatGPT-4, demonstrated superior diagnostic performance compared to dental students in endodontic assessments. The findings highlight the potential of AI as an educational support tool, particularly for reinforcing diagnostic standards. However, caution is advised regarding overreliance, which could hinder the development of students’ critical thinking and clinical decision-making skills. Further studies are needed to explore ethical and regulatory implications for broader implementation in dental education. Bias Risk: Low due to robust sample size, expert-defined gold standard, and appropriate statistical testing.
AI in Clinical, Diagnostic Training, and Radiographic Interpretation	Rampf et al., 2024, Germany [26]	To assess the impact of two feedback methods elaborated Feedback (eF) and Knowledge of Results (KOR) on radiographic diagnostic competencies in dental students, and to evaluate the diagnostic accuracy of an AI system (dentalXrai Pro 3.0) as a potential educational aid.	Randomized controlled trial (RCT). Fifty-five 4th-year dental students were randomly assigned to receive either eF or KOR while interpreting 16 virtual radiological cases over 8 weeks. Student diagnostic performance was assessed and compared, and the performance of the AI system was independently evaluated on the same tasks.	Randomized design with two intervention arms. Use of real radiographic cases and multiple diagnostic criteria (caries, apical periodontitis, image quality). Objective performance metrics (accuracy, sensitivity, specificity, ROC AUC). Inclusion of AI system benchmarking with near-perfect reported performance. Statistical comparisons conducted with appropriate tests (Welch’s t-test, ROC analysis).	Sample size: 55 students Some outcomes showed no significant differences between groups. Short duration (8 weeks) may not reflect long-term learning retention. No detailed analysis of how students interacted with AI or feedback content quality. No explicit control group without feedback or AI to isolate effects.	Students receiving elaborated feedback (eF) performed significantly better than those receiving only knowledge of results (KOR) in: Detecting enamel caries (↑ sensitivity, *p* = 0.037; ↑ AUC, *p* = 0.020) Detecting apical periodontitis (↑ accuracy, *p* = 0.011; ↑ sensitivity, *p* = 0.003; ↑ AUC, *p* = 0.001) Assessing periapical image quality (*p* = 0.031) No significant differences between groups were found for other diagnostic tasks. The AI system (dentalXrai Pro 3.0) showed near-perfect diagnostic performance: Enamel caries: Accuracy 96.4%, Sensitivity 85.7%, Specificity 7.4% Dentin caries: Accuracy 98.8%, Sensitivity 94.1%, Specificity 100% Overall: Accuracy 97.6%, Sensitivity 95.8%, Specificity 98.3%	Elaborated feedback significantly enhances diagnostic performance in selected radiographic categories compared to basic feedback. The AI system demonstrated near-perfect diagnostic capabilities, indicating strong potential as an alternative to expert-generated feedback in educational settings. However, the limited specificity in enamel caries AI performance (0.074) warrants cautious interpretation. The randomized design and direct AI benchmarking support the study’s reliability. Bias Risk: Low due to randomized controlled design, clear metrics, and direct AI-human comparison with transparent reporting.
AI in Clinical, Diagnostic Training, and Radiographic Interpretation	Schoenhof et al., 2024, Germany [27]	To investigate whether synthetic panoramic radiographs (syPRs), generated using GANs (StyleGAN2-ADA), can be reliably distinguished from real radiographs and evaluate their potential use in teaching, research, and clinical education	Experimental study with survey and test-retest reliability evaluation.	Included both medical professionals (*n* = 54) and dental students (*n* = 33). Used a controlled number of real (20), synthetic (20), and control (5) PRs. Assessed image interpretation accuracy, perceived image quality, and item agreement. Included test-retest reliability analysis.	Test-retest reliability was low (Cohen’s kappa = 0.23). Sample size modest (total *n* = 87), particularly within student subgroup (*n* = 33). Study used a limited set of images for evaluation (45 total).	Overall sensitivity for identifying synthetic images was 78.2%; specificity was 82.5%. Professionals: sensitivity 79.9%, specificity 82.3%. Students: sensitivity 75.5%, specificity 82.7%. Median image quality score: 6/10. Median rating for profession-related importance: 7/10. 11 out of 14 radiographic items showed agreement with expected interpretation.	The study demonstrates that GANs can generate highly realistic panoramic radiographs that are often indistinguishable from real ones by professionals and students. These synthetic images have educational and research value without privacy concerns. Bias Risk: Moderate due to small sample size, low retest reliability, and subjective evaluation metrics.
AI in Clinical, Diagnostic Training, and Radiographic Interpretation	Schropp et al., 2023, Denmark [28]	To evaluate whether training with AI software (AssistDent^®^) improves dental students’ ability to detect enamel-only proximal caries in bitewing radiographs and to assess whether proximal tooth overlap interferes with caries detection.	Randomized controlled study with two assessment sessions and reference-standard comparison.	Random allocation of 74 dental students to control and test groups. Use of validated software (AssistDent^®^). Two-session longitudinal structure allowed for measuring learning progression. Consideration of radiographic overlap as a variable influencing accuracy.	Only enamel-only caries assessed; may not generalize to more advanced lesions. Somewhat limited by moderate sample size (*n* = 74). AI assistance was only used in the first session	Session 1: No significant difference in positive agreement between control (48%) and test (42%) groups (*p* = 0.08). Test group had higher negative agreement (86% vs. 80%, *p* = 0.02). Session 2: No significant difference between groups. Within-group improvement: Test group improved in positive agreement over time (*p* < 0.001); control group improved in negative agreement (*p* < 0.001). Tooth overlap occurred in 38% of surfaces and significantly reduced diagnostic agreement (*p* < 0.001)	AI software did not significantly improve students’ diagnostic performance in detecting enamel-only proximal caries. However, both groups showed improvement over time. Tooth overlap negatively affected diagnostic accuracy regardless of AI use. Bias Risk: Low due to randomized design, adequate student sample, objective outcome comparison to reference standard.
AI in Clinical, Diagnostic Training, and Radiographic Interpretation	Suárez et al.,2022, Spain [29]	To evaluate dental students’ satisfaction and perceived usefulness after interacting with an AI-powered chatbot simulating a virtual patient (VP) to support development of diagnostic skills.	Descriptive cross-sectional study using a satisfaction survey following several weeks of interaction with the AI virtual patient.	Large and representative sample (*n* = 193, surpassing minimum of 169). Inclusion of students in two clinical years (4th and 5th year), allowing comparisons across experience levels. Gender-balanced data reporting. Integration of real student interaction with an AI tool over several weeks, enhancing ecological validity.	No control group; findings based solely on self-reported satisfaction rather than performance improvement. Cross-sectional design limits inference on causality or longitudinal impact. Outcomes focused on perception rather than objective diagnostic skill gain.	Overall high student satisfaction with the AI chatbot (mean score 4.36/5). Fifth-year students rated the tool more positively than fourth-year students. Students who arrived at the correct diagnosis through the chatbot interaction gave higher satisfaction ratings. Positive student perception supports the tool’s potential value for repeated diagnostic practice in a safe environment.	The AI-based virtual patient chatbot was well-received by students and viewed as a useful supplement for diagnostic training. It provided a cost-effective, space-saving educational solution that promotes engagement through natural language processing. Bias Risk: Moderate due to self-reported, perception-only data and absence of a comparator group, though the sample size and survey structure were strong.
AI as an Assessment Tool and Feedback System	Kavadella et al., 2024, Cyprus [30]	To evaluate the educational outcomes and student perceptions resulting from the real-life implementation of ChatGPT in an undergraduate dental radiology module using a mixed-methods approach.	Mixed-methods study involving a comparative learning task between two groups: one using ChatGPT and the other using traditional internet-based research. Data collection included knowledge exam scores and thematic analysis of student feedback.	Balanced sample size (*n* = 77) with well-structured group assignments. Combination of quantitative and qualitative data enhances depth of analysis. Statistical comparison of examination scores provides objective outcome data.	Small sample size per group (39 vs. 38), reducing generalizability. Short-term evaluation; no long-term retention or application assessment. Potential novelty bias influencing students’ enthusiasm for AI use. Single-institution study, limiting external applicability.	ChatGPT group outperformed traditional research group in knowledge exams (*p* = 0.045). Students highlighted benefits: fast response, user-friendly interface, broad knowledge access. Limitations identified: need for refined prompts, generic or inaccurate information, inability to provide visuals. Students expressed readiness to adopt ChatGPT in education, clinical practice, and research with appropriate guidance.	ChatGPT enhanced students’ performance in knowledge assessments and was positively received for its utility and adaptability in dental education. Students demonstrated critical awareness of its limitations and used it creatively. Bias Risk: Moderate due to short study duration, institution-specific sample, and self-reported feedback, although objective performance data strengthens credibility
AI as an Assessment Tool and Feedback System	Jayawardena et al., 2025, Sri Lanka [31]	To evaluate the effectiveness and perceived quality of feedback provided by an AI-based tool (ChatGPT-4) versus a human tutor on dental students’ histology assignments.	Comparative study analyzing 194 student responses to two histology questions. Feedback from ChatGPT-4 and human tutors was assessed using a standardized rubric. Students rated feedback on five dimensions, and an expert reviewed 40 randomly selected feedback samples.	Large sample size (*n* = 194) enhances reliability. Use of both student perception and expert evaluation provides multidimensional insight. Standardized rubric ensures consistency across assessments. Expert-blinded review of feedback enhances objectivity.	Focused only on histology questions; may not generalize to other areas of dentistry. Limited to one institution and subject area. Potential bias in student preferences due to familiarity with human feedback.	No significant score difference between AI and human feedback for one question; AI scored significantly higher for the second and overall scores. Students perceived no significant difference in understanding, critical thinking, relevance, or clarity, but preferred human feedback for comfort. Expert evaluation showed AI was superior in mistake identification, clarity (*p* < 0.001), and suggestions for improvement (*p* < 0.001).	ChatGPT-4 demonstrated effectiveness in providing clear and constructive feedback, performing comparably or better than human tutors based on expert analysis. Student preferences leaned toward human feedback due to emotional comfort, not quality. Bias Risk: Moderate while expert validation strengthens findings, the emotional bias in student responses and single-discipline scope limit broader generalization.
AI as an Assessment Tool and Feedback System	Ali et al., 2024, Qatar [32]	To explore the accuracy of ChatGPT in responding to various healthcare education assessment formats and discuss its implications for undergraduate dental education.	Exploratory study using 50 independently constructed questions spanning 5 common assessment formats (MCQs, SAQs, SEQs, true/false, fill-in-the-blank) and several academic writing tasks. Each format included 10 items. ChatGPT was used to attempt all questions.	Broad assessment coverage with 50 custom-developed items across multiple formats Realistic integration of AI use in student-assigned tasks Addresses both formative (e.g., feedback reports) and summative (e.g., MCQs) assessment types Explores qualitative and quantitative performance	ChatGPT’s inability to process image-based questions limits generalizability to clinical scenarios Only the free version of ChatGPT was tested Lack of benchmarking against student or educator performance Critical appraisal outputs from ChatGPT were found to be weaker	ChatGPT responded accurately to most knowledge-based question types (MCQs, SAQs, SEQs, true/false, fill-in-the-blank) ChatGPT struggled with image-based questions and critical appraisal tasks Generated reflective and research responses were mostly satisfactory Word count limitations noted with the free version	ChatGPT shows strong potential in supporting healthcare and dental education through accurate responses to diverse assessment types. However, its limitations in processing visual data and critical reasoning tasks highlight the need for educators to redesign assessments and learning approaches to integrate AI responsibly. Bias Risk: Moderate custom-designed questions ensure targeted evaluation, but lack of comparative analysis with human responses and reliance on only text-based questions limits generalizability.
AI in Content Generation for the Dental Field	Aldukhail et al., 2024, Saudi Arabia [33]	To evaluate and compare the performance of two large language models ChatGPT 3.5 and Google Bard in the context of dental education and research support.	Comparative evaluation using seven structured dental education-related queries assessed blindly by two reviewers. Scoring was based on pre-defined metrics and analysed using Wilcoxon tests for statistical significance.	Direct head-to-head comparison of two major LLMs in a dental education context Multi-domain evaluation covering exercises, simulations, literature critique, and tool generation Use of blind reviewers and statistical analysis to reduce subjective bias Practical relevance for educators seeking to integrate LLMs in curriculum design	Only ChatGPT 3.5 and Bard were tested, excluding newer or alternative models Evaluation was based on a limited number of prompts (*n* = 7), which may not generalise across broader contexts Reviewer subjectivity, despite blinding, could still influence scoring outcomes The study does not evaluate student learning outcomes following LLM use	ChatGPT 3.5 outperformed Google Bard in generating exercises, simulations, and assessment tools with higher clarity, accuracy, and specificity Google Bard showed strength in retrieving real research articles and critiquing them effectively Statistically significant differences (*p* ≤ 0.05) were found in scores for domains 1 (educational role) and 3 (simulations with treatment options) Both tools exhibited variability, highlighting the importance of user oversight in educational use	The study demonstrates that generative language models like ChatGPT and Bard can support dental education through simulation, content creation, and literature analysis. However, each model has distinct strengths and weaknesses, and critical judgment is essential when incorporating them into educational practice. Bias Risk: Moderate while the methodology includes blinding and structured scoring, the limited scope of prompts and model versions tested reduces the generalisability of findings.
AI in Content Generation for the Dental Field	Katebzad et al.,2024, USA [34]	To evaluate whether publicly available generative AI platforms can develop high-quality, standardized, and clinically relevant simulated pediatric dental cases for use in predoctoral education, including OSCE-style assessments. Pilot comparative study using standardized prompts across three de-identified AI platforms to generate pediatric dental cases on three themes. AI-generated cases were compared to investigator-generated (control) cases by two masked, board-certified evaluators using the AI-SMART rubric. Statistical analysis included ANOVA and Bonferroni correction.	Pilot comparative study using standardized prompts across three de-identified AI platforms to generate pediatric dental cases on three themes. AI-generated cases were compared to investigator-generated (control) cases by two masked, board-certified evaluators using the AI-SMART rubric. Statistical analysis included ANOVA and Bonferroni correction.	Use of standardized prompts ensures repeatability and consistency Evaluation performed by calibrated, blinded, board-certified examiners Introduces a novel rubric (AI-SMART) for systematic quality assessment Proof-of-concept for prompt engineering in dental education	Small sample size and pilot nature limits generalizability Control cases scored significantly higher, indicating AI limitations in clinical accuracy and OSCE formulation Some AI-generated answers conflicted with professional guidelines (e.g., AAPD) AI-SMART tool is not yet validated and requires further research Study did not assess student learning outcomes or implementation feasibility	No significant difference in clinical relevance (*p* = 0.44) and readability (*p* = 0.15) between AI and control cases Investigator-generated cases scored significantly higher in OSCE quality (*p* < 0.001) and answer accuracy (*p* = 0.001) AI platforms were efficient in producing interdisciplinary cases, but required manual review and correction Some AI-generated OSCE answers were incorrect or overly simplistic Highlighted the importance of standardized prompt design for effective AI use in education	AI platforms show promise for generating simulated pediatric dental cases efficiently, but human oversight and prompt engineering are essential to ensure clinical accuracy and alignment with professional guidelines. The study offers a valuable foundation for integrating AI in case-based learning, though further validation and broader testing are needed. Bias Risk: Moderate due to Using of blinded evaluators and statistical rigor adds reliability, but the tool used for scoring (AI-SMART) is unvalidated, and results are based on a pilot sample.

## 4. Discussion

This review confirms that AI holds promise in dental education, but highlights two major gaps limiting progress: the absence of a standardized definition of AI and the lack of clinician-led model development. Many studies misclassify tools like virtual reality as AI due to unclear definitions, undermining consistency across the field. Additionally, most AI tools are developed by computer scientists with minimal dental input, resulting in limited clinical relevance. By introducing a domain-based classification, this review offers a structured foundation that can help guide future studies toward more practical, targeted, and clinically meaningful AI applications in dental education.

### 4.1. AI in Preclinical Training

AI applications in this domain focus on skill development, simulation-based learning, and automated feedback systems to support students before they engage in direct patient care. Several studies have demonstrated AI’s effectiveness in improving procedural accuracy, reinforcing learning outcomes, and providing individualized feedback [18,19]. However, challenges remain in standardizing AI-driven training, ensuring reliability, and integrating AI assessments into competency-based education frameworks.

AI-driven simulation platforms have enhanced interactive preclinical training by providing structured, self-directed learning. Mahrous et al. [19] found that students using AI-generated feedback for prosthodontic design achieved higher accuracy than those with traditional instruction. Choi et al. [18] found AI useful in evaluating endodontic access cavity preparations. However, AI still struggles with assessing nuanced skills like hand dexterity, which require human oversight.

In the view of this scoping review’s authors, while AI offers measurable benefits in preclinical training, several challenges merit attention. While effective at assessing objective metrics like cavity depth, AI struggles with subjective skills such as dexterity and technique. Its accuracy depends on the quality and diversity of training datasets; any bias or limitation reduces reliability. Integrating AI into curricula also requires faculty training, infrastructure, and alignment. Moreover, excessive reliance on AI may hinder students’ development of self-assessment and critical thinking skills essential for clinical reasoning and growth.

### 4.2. AI in Clinical and Diagnostic Training and Radiographic Interpretation

AI is increasingly used in dental education to support clinical and diagnostic training, especially in radiographic interpretation and case-based reasoning. Studies by Qutieshat et al. [25], Rampf et al. [26], and Schropp et al. [28]. show that AI can improve diagnostic accuracy and standardize image interpretation, often outperforming student assessments in detecting caries, pulp, and periodontal pathologies. Similarly, Or et al. [20] reported improved diagnostic confidence from students using an AI chatbot for history-taking. These tools support real-time decision-making and diagnostic consistency, but over-reliance, ethical issues, and limited adaptability remain key barriers.

AI shows strong potential in radiographic interpretation, improving students’ ability to detect caries and other pathologies. Studies by Rampf et al. [26] and Qutieshat et al. [25] found that AI-enhanced diagnostic tools outperformed or matched student performance, especially in early enamel caries and endodontic cases. However, Schropp et al. [28]. highlighted concerns about AI model generalizability across systems, recommending AI as a supportive, not stand-alone tool. Additionally, Suárez et al. [29]. found AI chatbots improve students’ diagnostic reasoning, though Qutieshat et al. [25]. emphasized AI’s lack of clinical intuition, underscoring the continued need for human oversight.

In the view of the scoping review authors, while AI offers clear benefits in enhancing diagnostic skills, key challenges remain. There is a risk of students becoming overly reliant on AI, potentially diminishing their clinical reasoning and independent judgment. Ethical concerns around accountability also arise if AI-generated errors impact patient safety. Moreover, variability across AI models underscores the need for standardization and regulatory oversight in dental education.

### 4.3. AI as an Assessment Tool and Feedback System

AI is increasingly used in dental education for automated grading and real-time feedback, improving efficiency, reducing bias, and enhancing learning through personalized responses [31]. However, challenges remain, including difficulty evaluating complex answers, risks of student over-reliance, and concerns about transparency and bias.

AI-driven grading systems in dental education offer consistent and scalable assessment, improving efficiency and reducing variability [31]. However, students found AI feedback lacking in depth for open-ended and case-based tasks [32].

AI assessment tools provide real-time feedback that helps students self-correct and adapt [31]. In clinical skill evaluations, AI has improved performance by analyzing procedural accuracy and providing iterative feedback. However, proper calibration and faculty oversight are essential to prevent reinforcement of incorrect techniques.

In the view of the scoping review authors, while AI enhances assessment efficiency and consistency, key challenges persist. Students may become overly reliant on AI, potentially weakening their critical thinking. Current models struggle with nuanced evaluations involving communication and ethics. Additionally, privacy concerns and the lack of standardization across platforms limit broader integration into dental education.

### 4.4. AI in Content Generation for the Dental Education

AI is increasingly used in dental education to generate case-based materials, automated questions, and structured content. Tools like natural language processing and machine learning support the creation of curricular resources and adaptive learning modules [33,34]. These tools enhance efficiency and accessibility, but challenges persist around validating content, meeting accreditation standards, and avoiding bias.

AI has been used to create structured learning materials like case studies and assessments, enhancing diagnostic training and self-directed learning [34]. AI-powered search tools also outperform general models in retrieving accurate, relevant dental education resources [33].

AI has been used to automate the creation of MCQs and interactive assessments, helping generate quizzes aligned with key learning objectives [34]. This reduces faculty workload while supporting adaptive student practice.

In the view of the scoping review authors, despite its efficiency, AI-generated content in dental education presents key challenges. It requires careful review for accuracy and relevance, as limited training datasets may introduce bias. Content must also align with accreditation standards and competency frameworks, while remaining engaging enough to promote meaningful student learning and participation.

The advancement of this field is hindered by the absence of a standardized definition of AI within dental research, coupled with a notable shortage of input from dentists with AI expertise in the development of relevant platforms. Addressing these challenges through clinician-led AI research and the formulation of clearer definitions will significantly enhance the quality and relevance of future studies, paving the way for a more effective incorporation of AI in dental education.

### 4.5. Limitations

This review followed a rigorous PRISMA-ScR methodology; however, some relevant studies may have been missed due to database restrictions or keyword limitations [15] Many included studies had methodological constraints such as small sample sizes, unvalidated instruments, and limited generalizability [18,19,31] Furthermore, a reliance on student self-reported data introduces potential bias due to recall or social desirability factors [30]. Few studies explored faculty or curriculum designer perspectives, revealing a gap in understanding institutional readiness for AI integration [20,21,28]. Some methodological limitations should be acknowledged. The review was not registered in a protocol registry, and although multiple databases were searched, the inclusion was limited to English-language publications, which may have excluded relevant non-English studies. Additionally, no formal critical appraisal tool was used, and data extraction and bias assessment were conducted manually, which may introduce subjective interpretation despite independent review by two authors.

### 4.6. Recommendations for the Future

Addressing the lack of clinician-led AI development requires interdisciplinary collaboration between dental educators, clinicians, computer scientists, and industry partners. These efforts can ensure AI tools align with clinical and educational needs. Embedding AI literacy into dental curricula and faculty development will further equip clinicians to co-develop relevant tools. Collaborating with existing AI companies may also accelerate adaptation for dental training. Future research should also explore the development of AI tools that assess manual skills in preclinical simulation environments. Targeting core procedures such as cavity preparation, these tools can deliver high educational value by providing objective, real-time feedback aligned with expert standards. Such innovations could enhance consistency in preclinical assessment, support early skill acquisition, and reduce faculty burden, making them a valuable addition to dental education frameworks.

Ethical integration of AI in dental education demands more than identifying risks; it requires clear, actionable strategies. According to the World Health Organization’s guidance on AI ethics in health [35], responsible AI deployment must be grounded in principles such as transparency, accountability, inclusiveness, and data protection. Data privacy must be protected through secure systems and institutional governance frameworks. To minimize algorithmic bias, AI models should be trained on diverse datasets and validated with input from clinicians and educators. Clear accountability mechanisms must be established to address AI-generated errors. Educators should also take active steps to prevent student over-reliance on AI by embedding digital ethics into the curriculum, while fostering critical thinking and clinical judgment. These measures are essential to ensure that AI supports and complements rather than replaces core educational values.

Successful integration of AI into dental education depends not only on technological capability but also on faculty readiness and institutional support. Institutions must invest in professional development, cross-disciplinary collaboration, and infrastructure to prepare educators for the responsible adoption of emerging technologies. Without addressing these foundational needs, even well-designed AI tools may struggle to achieve meaningful implementation or acceptance. A balanced approach is needed, one that fosters innovation while safeguarding the quality of learning and maintaining the role of clinical judgment in education.

## 5. Conclusions

AI holds significant potential to enhance dental education, particularly in preclinical assessments where students develop foundational clinical skills. Its integration can support real-time feedback, improve diagnostic training, and personalize learning. However, current research remains limited, especially in evaluating core tasks such as restorative cavity preparation. Clear definitions of AI, standardized classification frameworks, and robust ethical oversight are essential to support consistent evaluation and safe implementation. At the institutional and policy level, integration of validated AI systems should be accompanied by clear governance structures, while educators should ensure that AI complements rather than replaces human oversight and judgment in student learning.

Moving forward, clinician-led AI development supported by education, research collaboration, and strategic investment will be essential to translating AI’s potential into meaningful, ethical, and context-aware advancements in dental education. To ensure relevance and reliability, future research should focus on developing real-time assessment tools grounded in clinical practice and led by dental educators. In particular, AI tools that evaluate manual skills in preclinical simulations should be prioritized, as they provide high educational value through expert-aligned, objective feedback that supports early skill acquisition and consistent assessment.

## Data Availability

The original contributions presented in this study are included in the article. Further inquiries can be directed to the corresponding author.
